# Interaction of class III cellobiose dehydrogenase with lytic polysaccharide monooxygenase

**DOI:** 10.1002/2211-5463.70067

**Published:** 2025-06-18

**Authors:** Angela Giorgianni, Florian Csarman, Peicheng Sun, Mirjam Kabel, Roland Ludwig

**Affiliations:** ^1^ Department of Biotechnology and Food Science, Institute of Food Technology BOKU University Vienna Austria; ^2^ Laboratory of Food Chemistry Wageningen University & Research The Netherlands

**Keywords:** cellobiose dehydrogenase, cyclic cascade reaction, electron transfer, hydrogen peroxide, lytic polysaccharide monooxygenase, rotating disk electrode

## Abstract

The genome of *Fusarium solani*, a well‐known plant pathogen, encodes various lytic polysaccharide monooxygenases (LPMOs) involved in plant biomass degradation in combination with cellobiose dehydrogenase (CDH). To investigate the auxiliary role of the recently expressed and characterized class III CDH from *F. solani* (*Fs*CDH), this enzyme was tested in combination with the well‐characterized AA9C from *Neurospora crassa* (*Nc*AA9C). Steady‐state and stopped‐flow methods as well as electrochemical measurements demonstrate how *Fs*CDH efficiently transfers electrons to *Nc*AA9C, with a rapid, observed heme reoxidation rate constant of 129 s^−1^. In comparison to ascomycete class II CDHs, the H_2_O_2_ production by *Fs*CDH is insufficient to promote LPMO activity. However, a cyclic cascade between *Nc*AA9C and *Fs*CDH was found. *Nc*AA9C reaction products showed a high catalytic efficiency as *Fs*CDH substrates, with *K*_M_ values close to its natural substrate cellobiose. This reaction was further investigated by a real time measurement, where *Fs*CDH and *Nc*AA9C were incubated with phosphoric acid‐swollen cellulose and the reaction was sustained over a long period without the addition of an external reductant. The new class III CDH is similar to other CDH classes, except its very low reactivity with molecular oxygen, pointing towards a different function in Ascomycota than class II CDH. These findings contribute to the better understanding of oxidative cellulose degradation by fungi and thus, to potential biotechnological applications for the sustainable use of biomass.

## Graphical abstract

The activity of lytic polysaccharide monooxygenase (LPMO) is supported by its auxiliary enzyme cellobiose dehydrogenase (CDH). The catalytic activity of both enzymes is coupled by electron transfer and a cyclic cascade generating substrates for both enzymes – hydrogen peroxide for LPMO and oxidized and non‐oxidized cellobiose and cello‐oligosaccharide species for CDH.

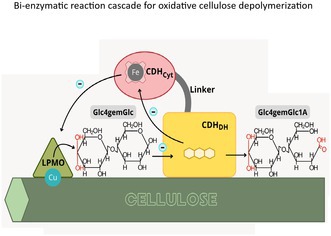

**Abbreviations**
AA: auxiliary activity
CDH: cellobiose dehydrogenase
*Fs*CDH: cellobiose dehydrogenase from *Fusarium solani*
LPMO: lytic polysaccharide monooxygenase
*Nc*AA9C: lytic polysaccharide monooxygenase auxiliary activity family 9 from *Neurospora crassa*
PASC: phosphoric acid‐swollen cellulose
RDE: rotating disc electrode

Plant cell wall constituents like cellulose and hemicellulose are abundant sources of carbohydrates, but their complexity makes depolymerization and extraction difficult [1, 2, 3]. Fungi are effective degraders of lignocellulosic biomass, with several interconnected metabolic strategies involving a plethora of secreted enzymes. Among these, CDHs and LPMOs operate together to enhance the degradation of the cellulosic fraction of plant biomass [4]. Among these enzymes, lytic polysaccharide monooxygenase (LPMO, EC 1.14.99.53–1.14.99.56, CAZy: AA9–11, AA13–17) catalyzes the oxidative cleavage of various polysaccharides. The reaction starts with the reduction of the enzyme's copper center by an electron donor, followed by the activation of the cosubstrate hydrogen peroxide (H_2_O_2_) at the copper center [3, 5, 6, 7, 8]. The catalytic reaction cleaves a glycosidic bond by inserting an oxygen atom either at the C1 or C4 carbon adjacent to it [6, 9, 10]. The auxiliary support of cellobiose dehydrogenase (CDH, EC 1.1.99.18, CAZy: AA3_1), a flavocytochrome enzyme present in fungal secretomes, towards LPMO was first noted for providing electrons to LPMO [4, 6]. Several studies have shown that CDHs interact with LPMOs from various fungal species, supporting the evolutionary conservation of this redox partnership [4, 11, 12]. Later, the production of H_2_O_2_ class II CDHs was also found to support the activity of LPMO [13, 14]. Compared to its dehydrogenase activity, the oxidase activity of CDH is low, with about 100 times lower turnover numbers for O_2_ than for quinoid electron acceptors. However, this low activity is suitable to boost LPMO activity [14]. In nature, the activity of LPMO needs to synchronize with the action of hydrolytic enzymes. The ability to regulate LPMO activity via its auxiliary enzyme CDH could permit fungi to tune oxidative depolymerization in accordance with hydrolytic activities according to biomass composition [6].

For this work, a preliminary genomic analysis of *Fusarium solani* (taxid: 169388) showed that a diverse set of putative *lpmo* genes was identified, including six from auxiliary activity (AA) family 9, five from family 11, one from family 13, two from family 14, and one from family 16. *F. solani* is a highly adaptable phytopathogenic fungus with worldwide distribution that infects a broad range of plant species, particularly targeting roots and causing soft rot in later stages of decay. Its host range includes numerous agriculturally significant crops such as legumes, potatoes, and cucurbits, reflecting its generalist pathogenic nature [15]. The extensive repertoire of LPMOs with a broad substrate spectrum likely plays a crucial role in the degradation of different plant cell wall polysaccharides, facilitating infection and nutrient acquisition [16]. The activation of these LPMOs can occur through different mechanisms [6] of which CDH is just one possibility.

The variability of fungal biomass degradation strategies is also reflected in the phylogenetic diversity of the large group of CDH enzymes. Previous phylogenetic analyses identified the presence of four distinct groups or classes within CDHs, some of which are also present in the same species [17, 18, 19, 20, 21]. Many class I and II CDHs have been characterized over time, with class I found only in basidiomycete fungi and class II from ascomycetes [19, 22, 23, 24, 25, 26, 27, 28, 29, 30]. In regard to class III CDHs also found in ascomycete genomes, many attempts have been made to produce an active member of this class until recently, when an active CDH class III from the ubiquitous plant pathogen *F. solani* was heterologously expressed and purified [21]. As investigated in previous work [21], sequence logos surrounding important catalytic residues showed that these are highly conserved across the three CDH classes. No class‐specific differences in these regions were detected, except for in the immediate vicinity of the catalytic His689 in class I *Phanerochaete chrysosporium* CDH (*Pc*CDH). In class I CDHs, Ser687 and Asn688 are strictly conserved, whereas in class II CDHs, Asn at the same position is strictly conserved, but the Ala (74.4%) can be replaced by Ser (24.4%). In contrast, in class III CDHs like the *F. solani* CDH, the position before the catalytic His is less conserved, with Ser663 (43.7%) found in a similar frequency as Asp (44.5%). Moreover, the position corresponding to the class I Ser687 is replaced by a highly conserved Gly in class III. Since the Ser687 of *Pc*CDH forms a hydrogen bond with the main polypeptide chain close to the substrate [24], a change at this position may not have a significant effect on substrate affinity or specificity. Nonetheless, the Asn688 residue directly engages in hydrogen bonding with the substrate in class I and II CDHs, but it is not present in class III CDH, possibly resulting in a varied substrate affinity or specificity. For both class I and II CDHs, the highest catalytic efficiency is reported towards cellobiose and cello‐oligosaccharides, while monosaccharides like glucose are poor substrates. Class II CDHs can convert a greater variety of mono‐ and oligosaccharides and exhibit less stringent discrimination against glucose than class I CDHs [31]. The preliminary characterization of *Fs*CDH showed a specific activity with cellobiose of 0.8 U·mg^−1^ at pH 6.0 using the DCIP assay [21]. This activity was lower compared to class I *Pc*CDH and class II *Mt*CDH (from *Myriococcum thermophilum*, syn. *Thermothelomyces myriococcoides*). The recombinantly expressed *Fs*CDH's low FAD occupancy (9%) was partly responsible for this decreased activity, and the recalculated activity for a 100% FAD occupancy is only marginally lower than that of class II *Mt*CDH under similar conditions [21]. The pH optimum for class I CDH is typically acidic, whereas some class II CDHs also perform under neutral or slightly alkaline conditions, which is probably an adaptation to the fungal lifestyle [31]. The so far tested conditions indicate a slightly acidic pH optimum of *Fs*CDH. This prompted us to use the well‐characterized AA9C from *Neurospora crassa* (*Nc*AA9C) to investigate the interaction of this newly characterized class III CDH with LPMOs. *Nc*AA9C has a similar pH optimum to *Fs*CDH, and in addition, *N. crassa* produces two well‐characterized class II CDHs with similarities to *Fs*CDH [21, 28].

This study investigates the auxiliary function of this newly characterized class III CDH on a well‐characterized *Nc*AA9C and investigates its electron transferring properties as well as its capability to produce H_2_O_2_, which has not been tested so far. This study also investigates a possible catalytic cascade between the depolymerizing and the auxiliary enzyme by testing LPMO reaction products as CDH substrates. The hypothesis to be tested was that when *Nc*AA9C cleaves cellulose, it produces a mixture of cellobiose and cello‐oligosaccharides as well as their C4‐oxidized forms [32] that are subsequently used by CDH as substrate. The reduced CDH would then be able to reduce the copper center of LPMO and thereby sustain the reaction cycle. *Fs*CDH, as the first characterized member of class III CDHs, is used as a representative to test if it can provide this auxiliary function as well as other CDH classes and support LPMOs in various fungi without class I and II CDHs. So far, oxidized cellulose breakdown products such as Glc4KGlc or its hydrated form Glc4gemGlc (nomenclature taken from Ref. [32]) have not been considered as substrates for CDH and will be tested in this study. When non‐oxidized and oxidized oligosaccharides produced by LPMO can serve as substrates for CDH, this cyclic cascade could be self‐maintained and possibly even self‐starting (Fig. 1). Studying the activity and interaction of both enzymes is at the focus of this work.

**Fig. 1:**
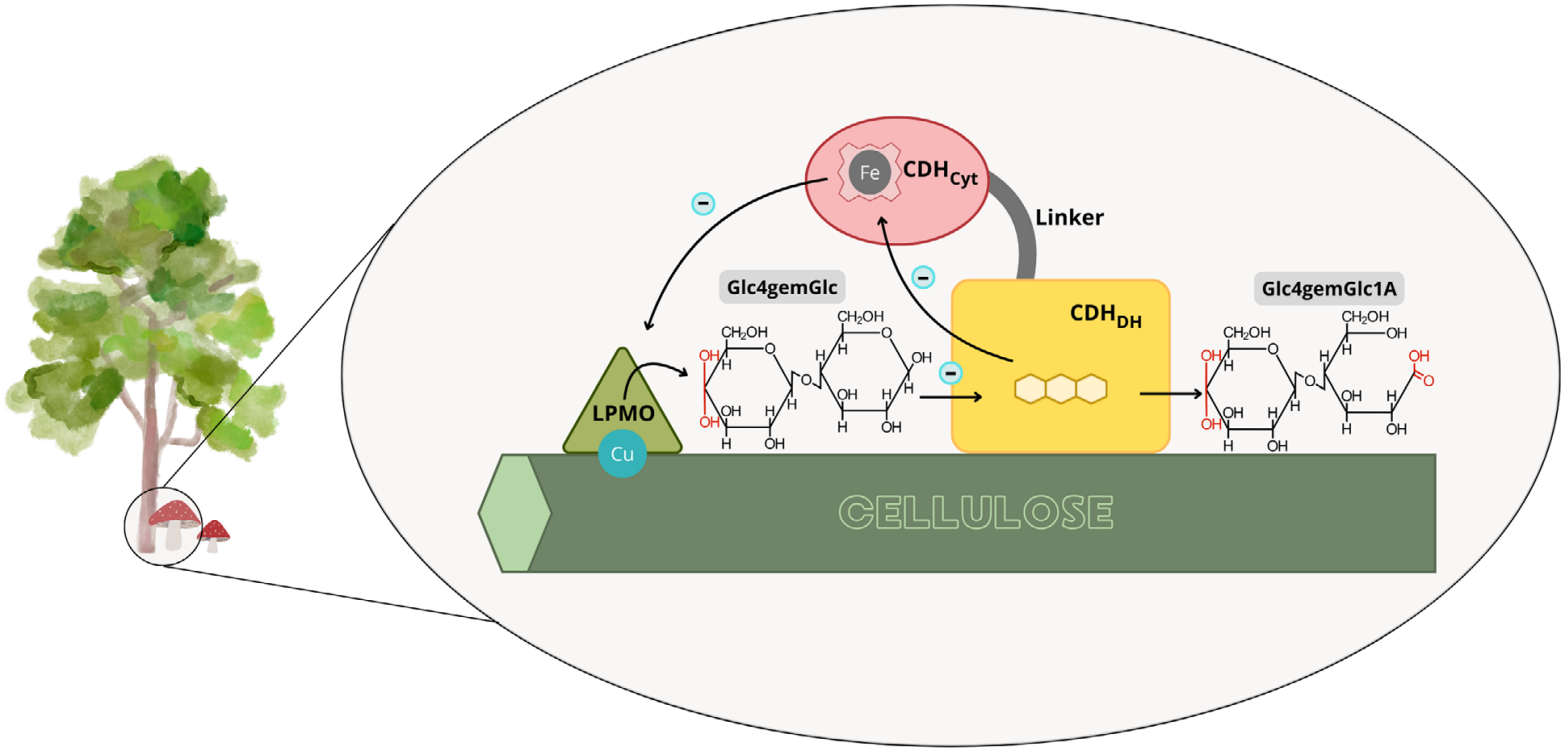
Schematic representation of the interaction between LPMO and CDH. Different types of LPMO produce oxidized or non‐oxidized cellulose degradation products, among which we find Glc4gemGlc, which could be a potential substrate to be oxidized by the dehydrogenase domain (CDH_DH_) of CDH. The electrons extracted during this reaction are transferred via the cytochrome domain (CDH_Cyt_) to external electron acceptors, such as lytic polysaccharide monooxygenases (LPMO). The production of H_2_O_2_ by CDH oxidizing carbohydrates instead of donating electrons to LPMO using O_2_ as an electron acceptor is not shown but occurs when CDH and LPMO are not in close contact.

## Materials and methods

### Chemicals, solutions, and enzymes

Buffer components and chemicals employed in this study were purchased at the highest available purity from Sigma (now Merck, Darmstadt, Germany), Fluka (Buchs, Switzerland), Roth (Karlsruhe, Germany), or VWR (Radnor, PA, USA). Microcrystalline cellulose (MCC) (*d* = 50 μm, 11365, Avicel PH‐101; Merck) was used to prepare phosphoric acid‐swollen cellulose (PASC) in accordance with a published protocol [33]. *Fs*CDH and *Nc*AA9C enzymes used in this investigation were heterologously produced in *Komagataella phaffii* syn. *Pichia pastoris* in 5‐L bioreactors and then purified by fast protein liquid chromatography (FPLC), as previously reported [21, 34].

### Stopped‐flow spectroscopy for *Fs*CDH‐LPMO interactions

Pre‐steady‐state kinetics measuring the reoxidation rate of *Fs*CDH by *Nc*AA9C were studied by stopped‐flow spectrophotometry (SX20; Applied Photophysics Ltd., Leatherhead, UK), as described previously [35]. A 1 : 1 molar ratio of 10 μm *Fs*CDH to cellobiose was incubated for 30 s to allow heme reduction and mixed rapidly with a 10 times higher concentration of 100 μm LPMO using the stopped‐flow sequential mode setup. The heme reoxidation of *Fs*CDH was monitored at 563 nm. The observed rate constants (*k*_obs_) were measured at pH 4.0, 5.0, and 6.0 in 50 mm sodium acetate buffer in quadruplet measurements.

### Evaluation of *Fs*CDH as a reducing agent for *Nc*AA9C

To determine if *Fs*CDH could act as a reducing agent for *Nc*AA9C, we monitored the H_2_O_2_ consumption in real‐time with a rotating disk electrode (RDE) setup [8]. The components of the reaction were: 30 mm sodium acetate buffer pH 5.0, 100 mm KCl, 4 g·L^−1^ xyloglucan as substrate for *Nc*AA9C, 0.2 μm *Nc*AA9C, varying concentrations (1–20 μm) of *Fs*CDH, and 1 mm cellobiose to reduce the cytochrome domain of *Fs*CDH. When all components except *Nc*AA9C were in the reaction cell, a calibration curve was measured by titrating increasing amounts of H_2_O_2_ until a final concentration of 100 μm was reached. Reactions were then started by adding *Nc*AA9C, and the initial rates for H_2_O_2_ consumption were measured under different concentrations of *Fs*CDH, as previously described [8]. The experimental error comprises the intraexperimental errors originating from the regression analysis for the determination of rate and the interexperimental errors originating from, for example, pipetting errors.

### RDE experiments for H_2_O_2_ production by *Fs*CDH

To monitor the production of H_2_O_2_ by *Fs*CDH, the previously mentioned RDE system was used in two different conditions. First, 2 μm *Fs*CDH was incubated in air‐saturated 30 mm sodium acetate buffer pH 5.0, with 100 mm KCl containing 10 mm cellobiose. H_2_O_2_ production was monitored using the RDE sensor, and the sensor calibration was performed before the end of the measurements by consecutive additions of 10 μm H_2_O_2_ aliquots up to a concentration of 50 μm. A second condition consisted of the incubation of 2 μm *Fs*CDH with 8 mg·mL^−1^ phosphoric acid‐swollen cellulose (PASC) dissolved in the same buffer. As a reference experiment, 0.2 μm *Nc*LPMO was added after 25 min, and after 30 min, 50 μm H_2_O_2_ was added as a cosubstrate for *Nc*AA9C to show that *Fs*CDH is active as an electron donor.

### RDE‐monitored batch reaction for *Nc*AA9C product generation

A batch reaction made it possible to generate *Nc*AA9C reaction products using 16 g·L^−1^ in 4 mL of cellopentaose as substrate and 5 mm ascorbic acid as reducing agent. The reaction was carried out in 30 mm sodium acetate buffer pH 5.0 supplemented with 100 mm KCl. *Nc*AA9C activity was measured in real time with the previously mentioned RDE setup [8]. H_2_O_2_ was titrated in 50 μm steps to replenish the cosubstrate of the reaction. Also, 1 μm *Nc*AA9C was periodically added to replace self‐inactivated LPMO activity. Complete substrate conversion was verified by the observation that subsequent additions of fresh NcAA9C aliquots no longer resulted in H_2_O_2_ consumption and by HPAEC‐PAD and MALDI‐TOF‐MS analysis on the products (Fig. S2).

### Purification and analysis of the C′4‐oxidized cellobiose

The naming of the C′4‐oxidized cellobiose is Glc4gemGlc and the C1‐oxidized Glc4gemGlc is termed Glc4gemGlc1A according to Isaksen *et al*. [32]. The reaction solution was firstly purified from enzymes and larger molecules through the use of Amicon Ultra Centrifugal Filters (Merck Millipore, Darmstadt, Germany) with a 10 kDa cut‐off. The flowthrough was then subjected to further purification by high‐performance size‐exclusion chromatography using polyacrylamide beads P‐2 Bio‐Gel (Biorad, Hercules, CA, USA) in an XK 16/100 column (Cytiva, Marlborough, MA, USA) equilibrated in water. The entire reaction volume was injected through a 2 mL injection loop in two steps. Fractions containing sugars were eluted with a flow of 0.5 mL·s^−1^, detected by monitoring the absorbance at 190 nm by the UV detector of the ÄKTA pure system (Cytiva), and collected. The selected fractions were then analyzed by thin‐layer chromatography for a first identification of the products (Fig. S3), with a solvent of *n*‐butanol/*n*‐propanol/ethanol/water mixture (2/3/3/2), following a standard procedure [36]. The fractions containing cellotriose were pooled, as well as fractions containing Glc4gemGlc, and purified separately, freeze‐dried, weighed, and redissolved in purified water for exact quantification. The identity of Glc4gemGlc was further confirmed by high‐performance anion‐exchange chromatography‐pulsed amperometric detection (HPAEC‐PAD) and matrix‐assisted laser desorption/ionization‐time of flight mass spectrometry (MALDI‐TOF‐MS) (Fig. S2). HPAEC analysis was performed with an ICS‐6000 system (Dionex, Sunnyvale, CA, USA) equipped with a CarboPac PA‐1 column (2 mm ID × 250 mm; Dionex) and a CarboPac PA guard column (2 mm ID × 50 mm; Dionex). 0.1 m sodium hydroxide (NaOH) and 1 m sodium acetate in 0.1 m NaOH were used as mobile phases. The elution method was performed as described previously [37]. The HPAEC‐PAD data was processed using Chromeleon 7.3.1 (Thermo Fisher Scientific, Waltham, MA, USA). MALDI‐TOF‐MS analysis was carried out with an Autoflex maX LRF system (Bruker Daltonics, Billerica, MA, USA). The instrument setup and sample preparation method were described previously [38]. The MALDI‐TOF‐MS data was processed using FlexAnalysis 3.4 (Bruker Daltonics).

### Steady‐state kinetics of *Fs*CDH using ketosugars as substrate

A portion of Glc4gemGlc was subjected to treatment with a commercial β‐glucosidase from almond seeds (Sigma‐Aldrich, now Merck, Darmstadt, Germany) for the degradation of possible non‐oxidized cello‐oligosaccharides (Glc4gemGlc‐treated). β‐glucosidase 2.5 U·mL^−1^ stock solution in 50 mm potassium phosphate buffer pH 6.0 was prepared. Subsequently, 200 μL β‐glucosidase stock solution was mixed with 50 μL supernatant to a concentration of 2 U·mL^−1^. The reaction was incubated in an Eppendorf Thermomixer at 800 r.p.m. at 37 °C for 24 h. The catalytic activity of *Fs*CDH was then assessed with both Glc4gemGlc and Glc4gemGlc‐treated samples using a standard 2,6‐dichlorophenol indophenol (DCIP, ε_520_ = 6.9 mm ^−1^·cm^−1^) assay. All assays were carried out in 50 mm potassium phosphate buffer pH 6.0, containing 0.3 mm DCIP and concentrations from 1 to 100 μm of Glc4gemGlc or Glc4gemGlc‐treated, from 1 μm to 3 mm for cellobiose and from 3 μm to 1 mm for cellotriose. All measurements were carried out in triplicates. Reduction of DCIP was monitored using an EnSpire Multimode plate reader (PerkinElmer, Waltham, MA, USA) for 3 min, and kinetic parameters were calculated from initial rates by fitting the data to the Michaelis–Menten equation using nonlinear least squares regression in sigmaplot 15.0 (Systat Software, San Jose, CA, USA).

### RDE‐monitored self‐sustaining reaction *Fs*CDH‐*Nc*AA9C system

The capacity of *Fs*CDH and *Nc*AA9C to sustain enzymatic plant biomass degradation in a self‐driven reaction system has been examined using the previously mentioned RDE system, with phosphoric acid‐swollen cellulose (PASC) as the substrate for *Nc*AA9C and only *Fs*CDH as its reducing agent. The reaction mixture contained 10 μm *Fs*CDH, 0.2 μm *Nc*LPMO9C, and 8 g·L^−1^ PASC in 30 mm sodium acetate buffer pH 5.0, with 100 mm KCl. H_2_O_2_ calibration for the used sensor was performed upfront and separately in a different reaction cell. The reaction was started by adding 50 μm H_2_O_2_ to the mixture. Additional titration of H_2_O_2_ fueled the system, and the addition of further aliquots of *Nc*AA9C counteracted enzyme auto‐inactivation. H_2_O_2_ consumption rates were calculated at each titration to assess the dynamics between *Fs*CDH and *Nc*AA9C.

## Results

### Interprotein electron transfer between *Fs*CDH and *Nc*AA9C

The reoxidation of the heme cofactor in *Fs*CDH's cytochrome domain by *Nc*AA9C was measured to investigate the interprotein electron transfer. The pre‐steady‐state rate of this reoxidation was studied by stopped‐flow spectrophotometry using the sequential mode under aerobic conditions. At the beginning of the experiment, the redox centers of CDH and LPMO were in their oxidized state. First, a molar ratio of *Fs*CDH and cellobiose was incubated for 30 s to allow heme reduction. The mixture was then shot against a 10‐fold more concentrated LPMO, and the reoxidation of the heme was followed at 563 nm. The results showed that *Fs*CDH was reduced during the incubation period of 30 s at pH 4.0 and 5.0, but barely at pH 6.0. This indicates a very slow interdomain electron transfer in *Fs*CDH at the highest tested pH value and removes this pH from the experimental conditions useful to study CDH‐LPMO interaction. The observed reoxidation rates at pH 4.0 and 5.0 were both very fast, with a *k*_obs_ value of 126.4 ± 16.0 s^−1^ and 129.3 ± 53.4 s^−1^, respectively.

### *Fs*CDH is able to effectively reduce *Nc*AA9C

**Fig. 2:**
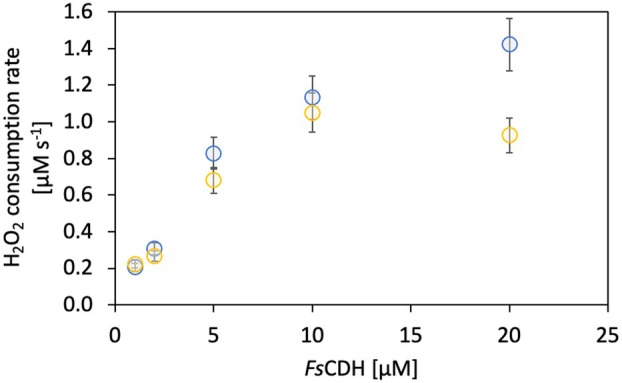
H_2_O_2_ consumption by *Nc*AA9C. Monitoring of *Nc*AA9C activity with varying concentrations of *Fs*CDH as a reducing agent. *Nc*AA9C's substrate consisted of 4 g·L^−1^ xyloglucan. The cytochrome domain of *Fs*CDH was reduced by incubating the enzyme with 1 mm cellobiose. The reaction was started by adding 0.2 μm *Nc*AA9C. Blue dots represent the rate calculated after the first titration with 100 μm H_2_O_2_, while the yellow data points were acquired after a second titration with 100 μm H_2_O_2_. The standard error of measurement (bars) comprises data from three technical replicates.

After characterizing the interprotein electron transfer between *Fs*CDH and *Nc*AA9C, we evaluated the efficiency of *Fs*CDH as an electron donor for *Nc*AA9C under steady‐state turnover conditions. To assess *Fs*CDH's saturating concentration for its interaction with *Nc*AA9C, we monitored H_2_O_2_ consumption by *Nc*AA9C after being reduced by *Fs*CDH. A rotating disk electrode (RDE) setup with a Prussian blue‐modified gold electrode was used for this purpose [8]. Initial consumption rates of H_2_O_2_ were measured for a fixed, 0.2 μm *Nc*AA9C concentration and varying (1–20 μm) concentrations of *Fs*CDH. The results show that there is a linear increase in the H_2_O_2_ consumption rate until 5 μm *Fs*CDH, reaching saturation at higher concentrations with an H_2_O_2_ consumption rate of 1.3 μm·s^−1^ at 20 μm *Fs*CDH (Fig. 2). The second titration of these experiments with 100 μm H_2_O_2_ showed the same results but revealed a decrease in the H_2_O_2_ consumption rate and therefore *Nc*AA9C activity, probably due to self‐inactivation [8].

### Hydrogen peroxide generation by *Fs*CDH

**Fig. 3:**
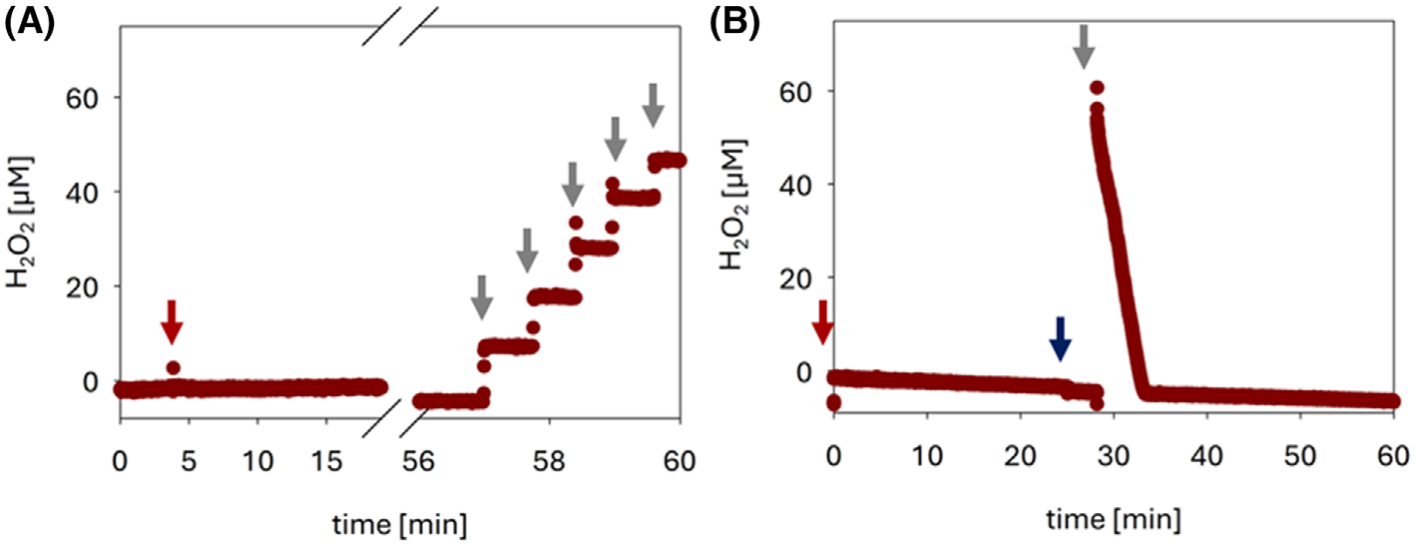
H_2_O_2_‐production by *Fs*CDH. (A) *Fs*CDH (2 μm, red arrow) was added to the air‐saturated buffer solution containing 10 mm cellobiose. No H_2_O_2_‐production was monitored using the RDE‐sensor. The sensor calibration was performed before the end of the measurements by consecutive additions of H_2_O_2_‐aliquots (10 μm per addition, gray arrows). (B) No detection of H_2_O_2_‐production by *Fs*CDH (2 μm, red arrow) in the presence of PASC (8 mg·mL^−1^). To verify the auxiliary function of *Fs*CDH, 0.2 μm *Nc*AA9C (blue arrow) was added after 25 min and 50 μm H_2_O_2_ after 30 min.

The capability of *Fs*CDH to utilize oxygen as an electron acceptor and thereby supply H_2_O_2_ to drive LPMO's peroxygenase activity was investigated using the RDE setup. The measurement was performed using either cellobiose or PASC as substrates for *Fs*CDH (Fig. 3). In both cases, no significant accumulation of H_2_O_2_ could be detected, indicating that *Fs*CDH does not produce H_2_O_2_ amounts detectable using the applied methodology. This is in contrast to the investigated class II CDHs from *M. thermophilum* or *N. crassa*. However, a control experiment (Fig. 3B) using PASC as a substrate showed that the addition of LPMO and H_2_O_2_ could initiate the catalytic conversion leading to the complete depletion of available H_2_O_2_ and verified the electron donating function of class III *Fs*CDH.

### Testing the cyclic cascade of *Fs*CDH and *Nc*AA9C

The cascadic conversion of *Nc*AA9C reaction products by *Fs*CDH was studied. The activity of *Fs*CDH towards the non‐oxidized reaction product cellobiose is established [21], but C4‐acting LPMOs like *Nc*AA9C also produce oxidized sugars. To study if an oxidized LPMO reaction product is a suitable substrate for CDH, a batch reaction employing *Nc*AA9C, H_2_O_2_ as cosubstrate, and ascorbic acid as a reducing agent was performed to convert 64 mg of cellopentaose. The reaction was followed by monitoring the H_2_O_2_ consumption by *Nc*AA9C with the RDE electrode setup, while stepwise titrating H_2_O_2_ to feed the reaction. To compensate for the self‐inactivation of the enzyme, successive additions of *Nc*AA9C were made until complete substrate conversion (Fig. S1). As previously found [32], the reaction produces a non‐oxidized cellotriose and the C′4‐oxidized Glc4gemGlc. The reaction products were then harvested and purified. HPAEC‐PAD analysis showed that Glc4gemGlc was the major component after purification, which is further confirmed by MALDI‐TOF‐MS analysis (Fig. S2).

### Non‐oxidized *Nc*AA9C reaction products are substrates for *Fs*CDH

Commercially available cellobiose and cellotriose were evaluated as substrates for *Fs*CDH using the standard DCIP assay, and steady‐state kinetic constants were determined (Table 1). For cellobiose, *Fs*CDH showed a *K*_M_ value of 21.4 μm, while a slightly lower *K*_M_ value of 16.1 μm was calculated for cellotriose. Both substrates show a similar catalytic efficiency (*k*_cat_/*K*_M_) of 0.06 and 0.07 μm ^−1^·s^−1^ respectively and demonstrate that non‐oxidized cellobiose and cello‐oligosaccharides are excellent substrates of the auxiliary action of *Fs*CDH.

**Table 1:** *Fs*CDH steady‐state kinetics measured with standard DCIP assay in a plate reader for cellobiose, cellotriose, Glc4gemGlc, and Glc4gemGlc after treatment with β‐glucosidase from almond seeds.

Substrate	*K*_M_ (μm)	*k*_cat_ (s^−1^)	*k*_cat_/*K*_M_ (μm ^−1^·s^−1^)
Cellobiose	21.4 ± 3.0	1.28 ± 0.03	0.06
Cellotriose	16.1 ± 2.9	1.08 ± 0.04	0.07
Glc4gemGlc	30.6 ± 8.3	1.91 ± 0.19	0.06
Glc4gemGlc‐treated	–	–	0.06

### Oxidized *Nc*AA9C reaction product is a substrate for *Fs*CDH

The *Nc*AA9C reaction product, Glc4gemGlc, was evaluated as a substrate for *Fs*CDH using the same standard DCIP assay. The Glc4gemGlc solution was used as is or after treatment with a commercial β‐glucosidase (Glc4gemGlc‐treated) to degrade any possible non‐oxidized cello‐oligosaccharides. The *K*_M_ value of *Fs*CDH for Glc4gemGlc is 30.6 ± 8.3 μm, indicating a good binding affinity for this substrate. The overall catalytic efficiency was 0.06 μm ^−1^·s^−1^. Due to the limited amount of substrate, we could not reach saturation for the Glc4gemGlc‐treated sample. Therefore, only the catalytic efficiency was calculated from the initial linear phase, with a similar result of 0.06 μm ^−1^·s^−1^. This shows for the first time that an oxidized LPMO reaction product such as Glc4gemGlc is an excellent substrate of a CDH, similar to cellobiose.

### CDH‐LPMO interaction can be self‐driven

**Fig. 4:**
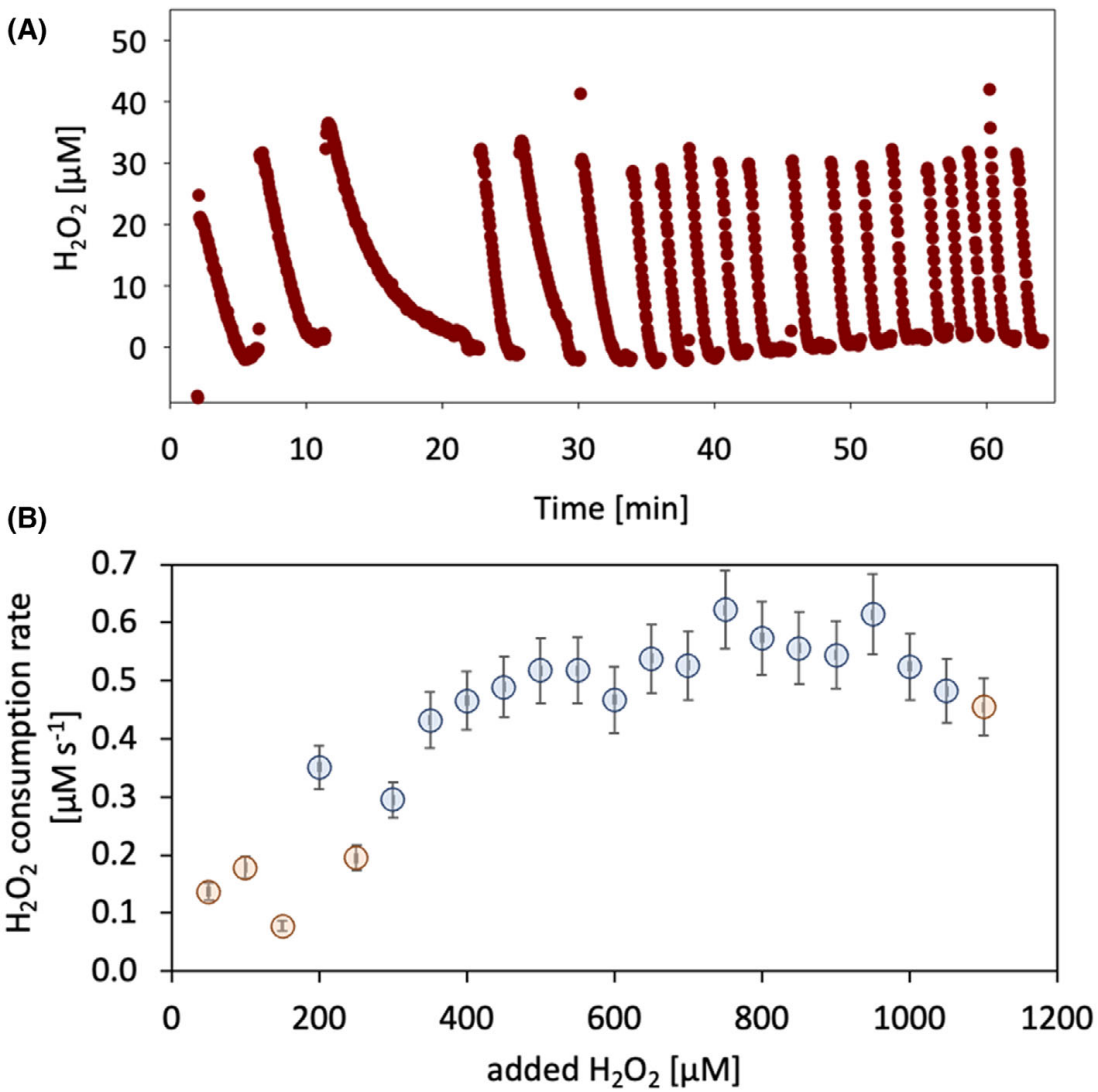
Real‐time monitoring of *Nc*AA9C H_2_O_2_ consumption with *Fs*CDH as electron donor. (A) H_2_O_2_ consumption of *Nc*AA9C monitored online by the RDE setup using a Prussian blue‐modified gold electrode and (B) calculated H_2_O_2_ consumption rate for the same experiment. The reaction mixture consisted of 10 μm *Fs*CDH as the electron donor for 0.2 μm *Nc*AA9C, 8 g·L^−1^ PASC as substrate for LPMO, in 30 mm sodium acetate buffer pH 5.0, 100 mm KCl. In this setup, *Fs*CDH used cello‐oligosaccharides present in the PASC mixture as substrates. The reaction was started by adding 50 μm H_2_O_2_. In (B) the red data points represent the rate calculated from measurements taken after the addition of 50 μm H_2_O_2_. Since a decreased rate was observed after a couple of titration points, more likely due to *Nc*AA9C auto‐inactivation, the blue data points represent the slope calculated from measurements taken after the addition of both 0.2 μm *Nc*AA9C and 50 μm H_2_O_2_. The standard error of measure (bars) comprises data from three technical replicates.

To test the cascadic interaction of *Fs*CDH and *Nc*AA9C over a prolonged time and confirm the self‐sustenance of this enzymatic system, the ability of *Fs*CDH to act as the sole reducing agent for *Nc*AA9C and the LPMO reaction products as substrates for the CDH, a batch reaction was performed. The setup consisted of a 4‐mL electrochemical cell to monitor the H_2_O_2_ consumption by LPMO in real time. Phosphoric acid‐swollen cellulose (PASC) was selected as the substrate for *Nc*AA9C, as the reducing ends of the cellulose chains should be accessible to CDH to quick‐start the reaction without the need for adding additional cellobiose. *Fs*CDH was the only electron donor present and was incubated together with *Nc*AA9C and PASC. The reaction was initiated by the addition of 50 μm H_2_O_2_ and further H_2_O_2_ titrations were performed when the previous addition was consumed by *Nc*AA9C (Fig. 4A). To compensate for *Nc*AA9C self‐inactivation, several subsequent H_2_O_2_ titration steps were accompanied by the addition of fresh aliquots of the enzyme. The H_2_O_2_ consumption rate for each titration step was calculated (Fig. 4B, Table S1), showing how the reaction can continue stably over time, until reaching a plateau of around 0.5 μm·s^−1^. This occurred without the addition of a di‐ or oligo‐saccharide substrate for *Fs*CDH. To further confirm the role of CDH as a necessary electron donor in this reaction, a control experiment was set up in the absence of *Fs*CDH, showing that *Nc*AA9C cannot efficiently consume H_2_O_2_ unless a reductant such as ascorbic acid was added (Fig. S4). This suggests that the initial reducing glucose ends of PASC and later on the *Nc*AA9C products are sufficient for the functioning of *Fs*CDH.

## Discussion

The aim of this work was to investigate the behavior of a member of the newly characterized class III *Fs*CDH in its catalytic interaction with the well‐known *Nc*AA9C [34]. Testing at first the electron transfer between the two enzymes, which has been the first discovered interaction between both enzymes [4, 6, 10], both stopped‐flow and RDE experiments confirmed that *Fs*CDH can efficiently act as an electron donor for *Nc*AA9C and thereby substitute small molecular reductants such as ascorbic acid, gallic acid, etc. Despite the different origin of the enzymes, the measured interprotein electron transfer rate is the highest measured so far [8]. *Fs*CDH's kinetic analysis revealed a similar behavior to class I *Pc*CDH, where cellobiose is also a preferred substrate, showing a *K*_M_ of 0.08 mm and a *k*_cat_ of 27.8 s^−1^, and to class II CDH (*Nc*CDH) with a *K*_M_ of 0.05 mm and a *k*_cat_ of 24 s^−1^ when tested in similar conditions [39, 40]. Pre‐steady‐state kinetic analysis shows that CDH's cytochrome domain gets rapidly re‐oxidized by *Nc*AA9C at pH 4.0 and 5.0. These results are in agreement with the previous studies, in which an interspecies CDH‐LPMO system was also studied, using a CDH from *Crassicarpon hotsonii* (syn. *M. thermophilum*) and the same LPMO employed in this study [41]. There, a second order rate of 9.87 × 10^5^ m ^−1^·s^−1^ was obtained at pH 4.5, which is a little lower than the one obtained in this study for *Fs*CDH‐*Nc*AA9C: about 1.3 × 10^6^ m ^−1^·s^−1^ for both pH 4.0 and 5.0. Interestingly, the interdomain electron transfer of *Fs*CDH at pH 6.0 was slower than the experimental time, which indicates a very slow interdomain electron transfer at near neutral conditions. This pH‐dependent behavior is similar to class I CDH from *P. chrysosporium* [42]. The RDE experiment confirmed that *Fs*CDH can effectively activate *Nc*AA9C at pH 5.0, the turnover numbers increasing with an increasing *Fs*CDH concentration, reaching saturation at 20 μm. This observation confirms the reported data for the *Nc*CDHIIA‐*Nc*AA9C enzyme system in the same setup [8] and demonstrates that CDH is a very efficient electron donor to LPMO.

Besides its function to supply electrons for the initial reduction of the LPMO copper redox center, CDH has been demonstrated to generate H_2_O_2_ through its oxidase side activity [3, 6, 14]. In a study by Chang *et al*. using an engineered CDH variant with increased oxygen reactivity, the accumulation of H_2_O_2_ with a substrate but in the absence of an electron acceptor except oxygen could be demonstrated using an amperometric sensor. However, in the presence of LPMO, the H_2_O_2_ accumulation was not observed, suggesting the immediate consumption of H_2_O_2_ by LPMO and confirming that the H_2_O_2_ production by CDH is the rate‐limiting step in the auxiliary activation of LPMO [7]. This study found that native *Fs*CDH has an extremely low reactivity towards oxygen and even in the absence of LPMO does not produce significant amounts of H_2_O_2_ when incubated with cellobiose or PASC. The results show that *Fs*CDH is supporting LPMO activity by direct electron transfer, but not by H_2_O_2_ generation or only at an extremely low, undetectable rate. This implicates that other, external H_2_O_2_ sources are required for driving *Nc*AA9C catalysis. However, the possibility of H_2_O_2_ production by LPMO in the presence of *Fs*CDH as a reductant cannot be excluded [34]. In any case, the fast peroxygenase activity of LPMO in the presence of a substrate prevents the accumulation of detectable amounts of H_2_O_2_ [3, 7].

Finally, this study investigated the cascadic use of substrates between both enzymes. *Nc*AA9C reaction products were prepared and performed perfectly as substrates for *Fs*CDH, thus establishing a catalytic cycle. Results showed that both non‐oxidized (cellobiose, cellotriose) and the oxidized Glc4gemGlc were efficient substrates for *Fs*CDH, reflected in the low *K*_M_ values of 21.4 ± 3.0 μm for cellobiose, 16.1 ± 2.9 μm for cellotriose, and 30.6 ± 8.3 μm for Glc4gemGlc. For the non‐oxidized substrates, this behavior is well‐known and not surprising. It is similar to class II *Nc*CDHIIA with a *K*_M_ of 0.05 mm and a *k*_cat_ of 24 s^−1^ for cellobiose when tested in similar conditions [40]. Surprisingly, the ability of *Fs*CDH to react with an oxidized sugar and the substrate configuration of Glc4gemGlc is still compatible with the CDH active site.

Taking into consideration all the aspects discussed above, a final RDE experiment was performed to obtain an overview of the interaction between *Fs*CDH and *Nc*AA9C over prolonged turnover conditions. In this reaction, *Fs*CDH was the only source for electrons, and the degradation of PASC by *Nc*AA9C provided the substrate for the CDH. The addition of the LPMO cosubstrate H_2_O_2_ in discrete titration steps is necessary since it is not or only insufficiently produced by *Fs*CDH. The very low H_2_O_2_ production rate by *Fs*CDH is, however, also a benefit, allowing the exact measurement of H_2_O_2_ turnover by *Nc*AA9C. The titration with H_2_O_2_ resulted in a relatively high 50 μm H_2_O_2_ concentration, leading to a fast deactivation of the *Nc*AA9C, as shown in previous studies [8, 14]. The observed stable H_2_O_2_ consumption supports the hypothesis that CDH and LPMO can form a self‐sustaining enzymatic cascade in which LPMO‐generated depolymerization products act as CDH substrates, which in turn boost the oxidative degradation cycle in a mechanism that might resemble fungal lignocellulose degradation in physiological conditions.

In this study, we investigated the interaction between the newly characterized *Fs*CDH, belonging to a newly characterized CDH class together with the well‐known *Nc*AA9C to explore their interaction. Overall, our findings advance the understanding of the complex mechanistic routes involved in oxidative cellulose degradation by fungi, which concerns not only ecological implications but also biotechnological applications for the sustainable use of biomass.

## Conflict of interest

The authors declare no conflict of interest.

## Author contributions

RL contributed to conceptualization; AG, FC, and PS contributed to methodology, investigation, and visualization; FC and MK contributed to formal analysis; RL and MK contributed to resources; AG contributed to data curation and writing—original draft preparation; FC, PS, MK, and RL contributed to writing—review and editing; MK and RL contributed to supervision; RL contributed to project administration and funding acquisition. All authors have read and agreed to the published version of the manuscript.

## Supporting information


**Fig. S1.** Production of *Nc*AA9C reaction products.
**Fig. S2.** Confirmation of the *Nc*AA9C reaction product Glc4gemGlc.
**Fig. S3.** TLC analysis of *Nc*AA9C reaction products.
**Fig. S4.** RDE control experiment.
**Table S1.** Titration details.

## Data Availability

All data generated or analyzed in the course of this study have been included in the manuscript or Supporting Information files.
