# Supplementary material for: Interaction of class III cellobiose dehydrogenase with lytic polysaccharide monooxygenase
Source: FEBS Open Bio. 2025 Jun 18;15(10):1618–28. doi: 10.1002/2211-5463.70067 (PMC12485664; doi:10.1002/2211-5463.70067)
Supplement: Supplementary file 1 — Fig. S1. Production of NcAA9C reaction products. Fig. S2. Confirmation of the NcAA9C reaction product Glc4gemGlc. Fig. S3. TLC analysis of NcAA9C reaction products. Fig. S4. RDE control experiment. Table S1. Titration details. [file FEB4-15-1618-s001.pdf]

Supplementary Materials

Interaction of class III CDH with LPMO

Angela Giorgianni[1], Florian Csarman[1], Peicheng Sun[2], Mirjam Kabel[2], Roland Ludwig[1*]

[1] Institute of Food Technology, Department of Biotechnology and Food Science, BOKU University, Muthgasse 18, 1190 Vienna, Austria

[2] Laboratory of Food Chemistry, Wageningen University & Research, Bornse Weilanden 9, 6708 WG Wageningen, The Netherlands

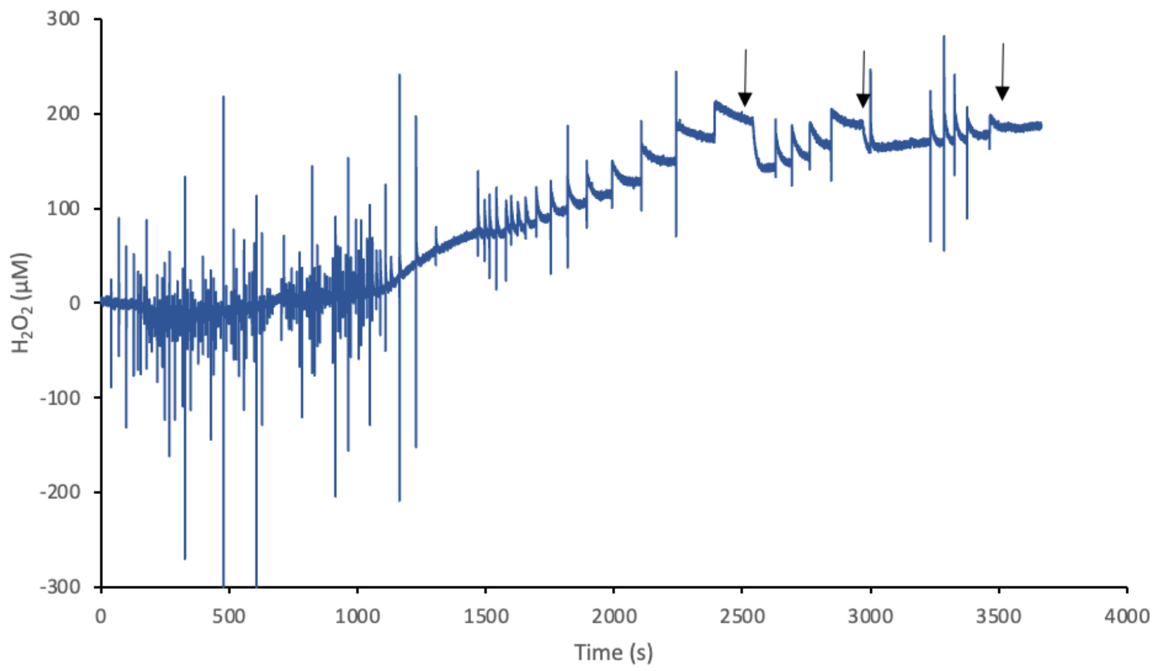

Figure S1. Production of *Nc*AA9C reaction products. The H_2O_2 concentration during the batch conversion of 16 g L^{-1} of cellopentaose in 30 mM sodium acetate buffer, pH 5.0, containing 100 mM KCl was monitored with an RDE electrochemical cell. *Nc*AA9C activity quickly consumes the titrated H_2O_2. Arrows represent an additional 1 μM *Nc*AA9C supplementation to replace the inactivated enzyme and to reach full conversion of the substrate.

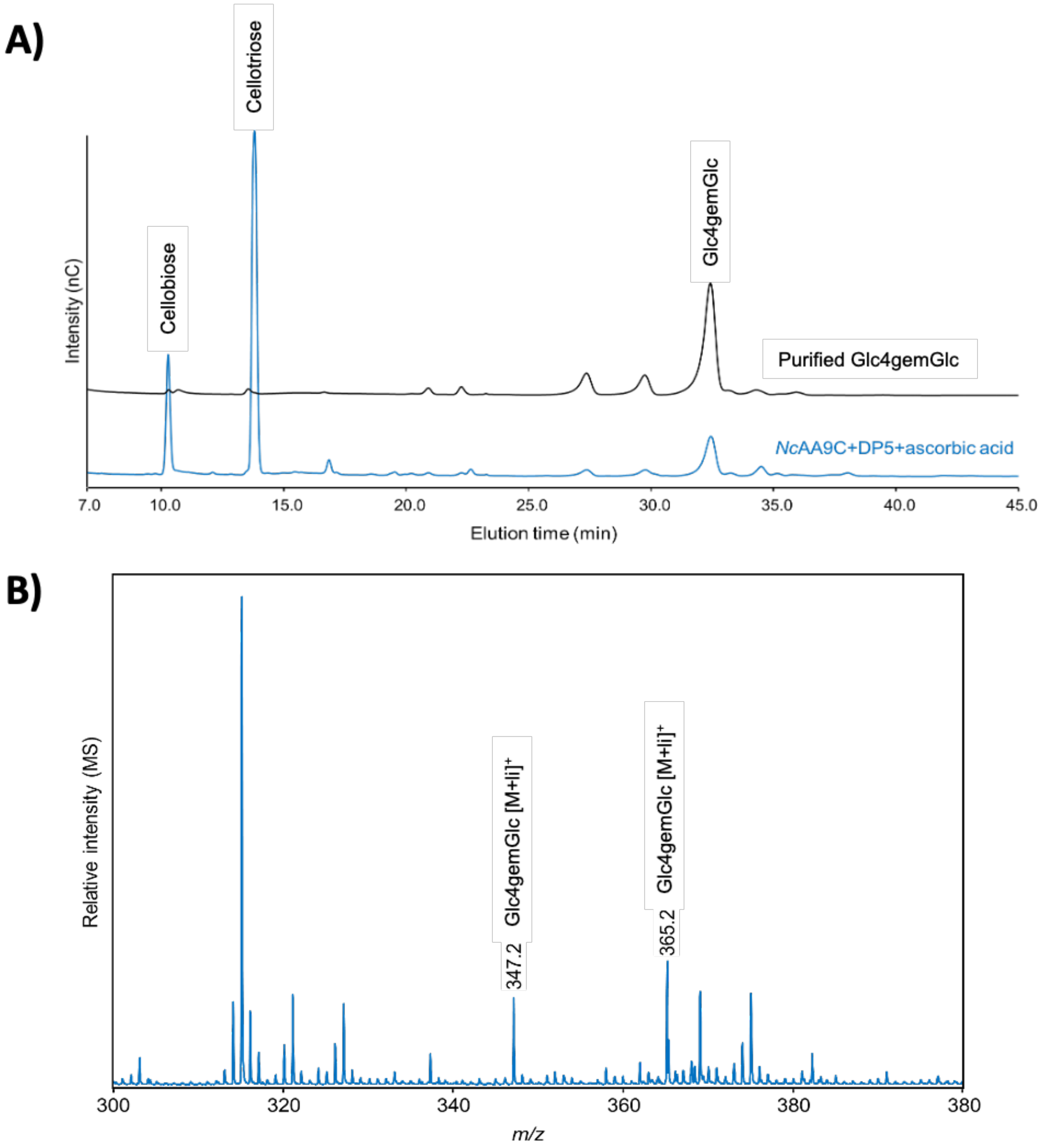

Figure S2. **Confirmation of the *Nc*AA9C reaction product Glc4gemGlc.** A) HPAEC-PAD analysis and B) MALDI-TOF-MS analysis both confirmed the main presence of Glc4gemGlc (peak labeled as DP2ox), with contaminants derived from the reaction mixture such as ascorbic acid derivatives and small amount of non-oxidized cello-oligosaccharides (cellotriose labelled as DP3). In MALDI-TOF-MS, Glc4gemGlc presents both, the 4-ketoaldose (*m/z* 347.2) and the gem-diol (*m/z* 365.2) forms.

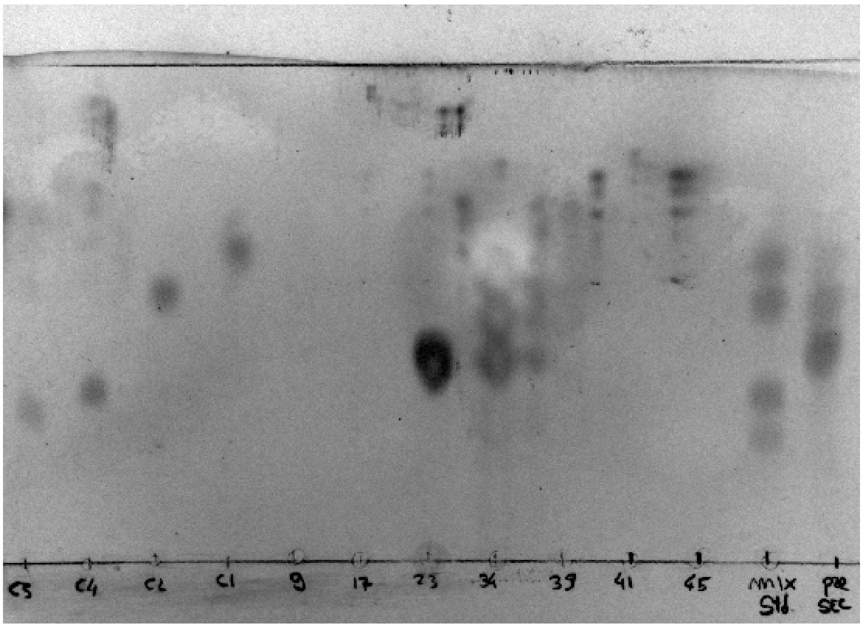

Figure S3. **TLC analysis of NcAA9C reaction products.** TLC for a first evaluation of fractionated samples from *Nc*AA9C's reaction products after SEC purification. The evaluated samples from left to right are standard cellopentaose; standard cellotetraose; standard cellobiose; standard glucose; fraction number 9; fraction number 17; fraction number 23; fraction number 34; fraction number 39; fraction number 41; fraction number 45; mix of cellopentaose, cellotetraose, cellobiose and glucose; sample of NcAA9C reaction products before SEC separation. Fractions were chosen based on the peaks shown in the chromatogram during separation.

Table S1. Titration details. Details for each titration step performed during the RDE experiment shown in Figure 4.

Titration content	Time addition (s)	rate (μM s^{-1})	added H_2O_2 (μM)
50 μM H_2O_2	130	0.14	50
	400	0.18	100
	700	0.08	150
	1550	0.20	250
	3930	0.45	1100
50 μM H_2O_2 + 0.2 μM LPMO	1370	0.35	200
	1820	0.29	300
	2040	0.43	350
	2170	0.47	400
	2290	0.49	450
	2430	0.52	500
	2550	0.52	550
	2750	0.47	600
	2900	0.54	650
	3050	0.53	700
	3180	0.62	750
	3340	0.57	800
	3440	0.56	850
	3530	0.54	900
	3610	0.61	950
	3730	0.52	1000
	3840	0.48	1050

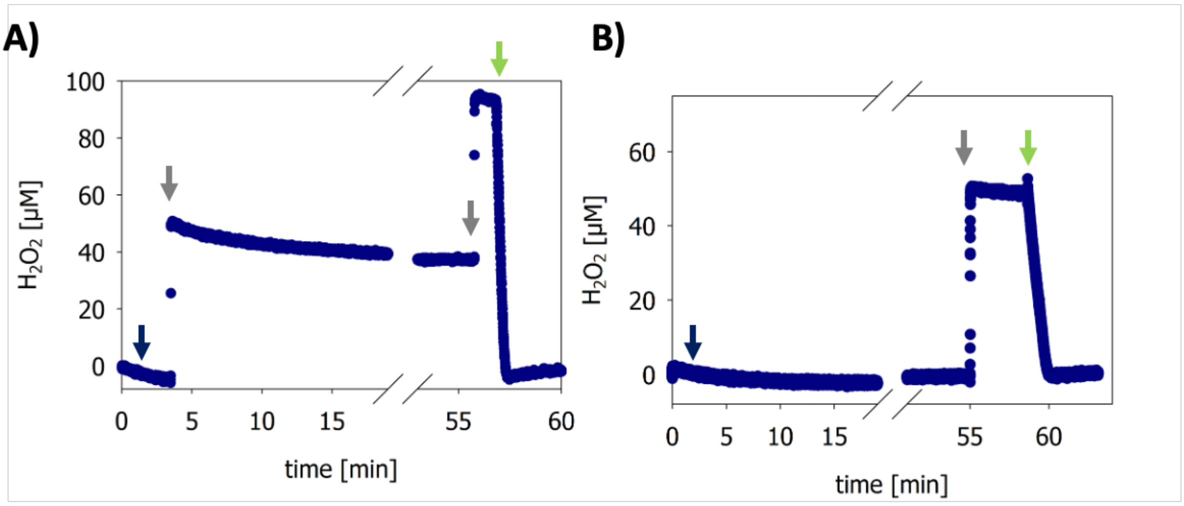

Figure S4. RDE control experiment. Determination of whether *Nc*AA9C without *Fs*CDH can consume H_2O_2. The reaction solution consisted of 30 mM sodium acetate buffer, pH 5.0, with 100 mM KCl, in the presence of 8 mg mL^{-1} phosphoric acid-swollen cellulose (PASC). Two cases were tested and monitored over 1 h: **A**) incubation of *Nc*AA9C (blue arrow) in the presence of 50 µM H_2O_2 (grey arrow); **B**) incubation of *Nc*AA9C (blue arrow) without H_2O_2. In both cases, after 55 min a further 50 µM H_2O_2 aliquot and 500 µM ascorbic acid (green arrow) were added, to verify the efficient consumption of H_2O_2 by *Nc*AA9C only in the presence of a reducing agent, like ascorbic acid.
